# AI-assisted quantitative analysis for evaluating melanin distribution in 3D pigmented epidermis-on-a-chip models

**DOI:** 10.3389/fbioe.2026.1787959

**Published:** 2026-04-07

**Authors:** Yu Yao, Xuan Du, Yanhui Li, Yuchen Ma, Yuchen Li, Zilin Zhang, Boyang Song, Xiaoran Li, Jing Zhang, Jun Ouyang, Nuo Si, Ningbei Yin, Qianqian Han, Zhongze Gu, Zaozao Chen

**Affiliations:** 1 Plastic Surgery Hospital, Chinese Academy of Medical Sciences and Peking Union Medical College, Beijing, China; 2 Skin-on-a-Chip Translational Medicine Center, Joint Laboratory of Plastic Surgery Hospital, Chinese Academy of Medical Sciences and Southeast University, Beijing, China; 3 State Key Laboratory for Novel Software Technology, Nanjing University, Nanjing, China; 4 State Key Laboratory of Digital Medical Engineering, Institute of Microphysiological Systems, School of Biological Science and Medical Engineering, Southeast University, Nanjing, China; 5 National Institutes for Food and Drug Control, Beijing, China

**Keywords:** AI-based quantitative evaluation, melanin distribution, pigmented epidermis-on-a-chip, semantic segmentation, vision transformer (ViT)

## Abstract

**Introduction:**

Abnormal pigmentation plays an important role in various skin diseases and in studies of whitening efficacy.Three-dimensional pigmented epidermis-on-a-chip models provide a crucial in vitro platform for exploring melanin production and regulation in skin. However, dynamic and non-invasive quantitative assessment of melanin distribution remains difficult with traditional histological methods.

**Methods:**

In this study, an AI-assisted objective evaluation framework was established for three-dimensional pigmented epidermis-on-a-chip models based on brightfield images. Melanin regions were segmented using the MEM-ViT algorithm, and their morphological features were extracted to build a multi-indicator comprehensive analysis system for determining the “good/poor” status of the model.

**Results:**

The results showed 98% consistency between algorithmic predictions and manual annotations, demonstrating the reliability and generalization capability of the proposed method. The framework enabled accurate segmentation of melanin regions and standardized evaluation of model quality without staining.

**Discussion:**

This method provides a rapid, non-invasive, and standardized approach for evaluating 3D pigmented epidermis-on-a-chip models. It offers a useful technical pathway for drug efficacy research, whitening mechanism analysis, and objective assessment of skin pigmentation-related disorders.

## 1 Introduction

**FIGURE 1:**
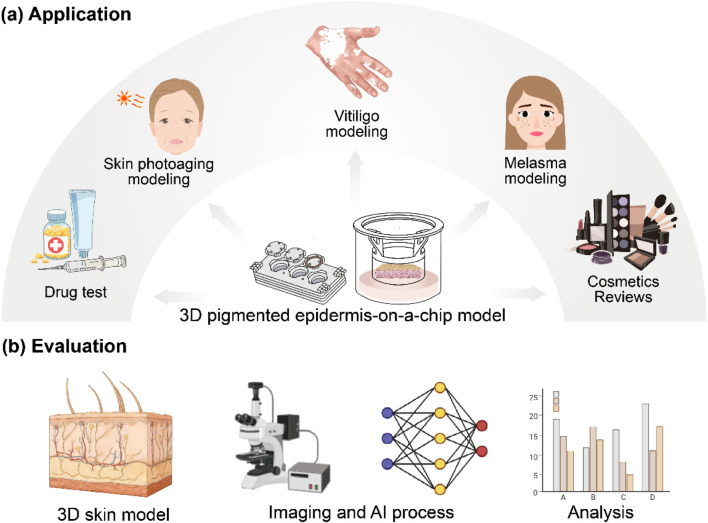
Applications of the pigmented epidermis model and AI-based evaluation workflow: **(a)** Model applications: Pigmented epidermis-on-a-chip models are used for disease modeling of photoaging, vitiligo, melasma, and related pigmentary disorders, and efficacy testing of drugs and cosmetic products. **(b)** AI evaluation workflow: Brightfield images acquired from 3D pigmented epidermis-on-a-chip models are processed by image preprocessing and segmentation using the MEM-ViT algorithm, followed by extraction of quantitative metrics and statistical analysis.

Pigmentary disorders (e.g., melasma, cutaneous photoaging, and marginal repigmentation in vitiligo), and efficacy evaluation of cosmetic products including skin-whitening/spot-lightening and photoprotective skincare, urgently require an in vitro model to recapitulate both melanogenesis and intercellular melanin transfer, while capturing the spatial distribution and temporal dynamics of pigmentation (Figure 1a) (Lei and Hearing, 2020; Bento-Lopes et al., 2023; Miao et al., 2025). Conventional two-dimensional monolayer cell models cannot couple melanocytes and keratinocytes and lack the stratified barrier context (Costa Gagosian et al., 2025; Seiberg, 2001). This makes it difficult to reproduce clinically relevant spatial phenotypes such as pigment homogeneity, lesion area, and pigment deposition. Therefore, *in vitro* experimental results often show poor correlation with clinical efficacy. In contrast, three-dimensional pigmented epidermis-on-a-chip models are co-constructed from melanocytes and keratinocytes (Li et al., 2023). In addition, they possess a stratified architecture from the basal layer to the stratum corneum, as well as paracrine signaling, cell–cell adhesion, and receptor-mediated pathways between neighboring cells (Hall et al., 2022; Tada et al., 1998; Correia et al., 2018). These features confer higher external validity for drug screening, efficacy verification, and safety assessment.

Traditionally, skin model evaluation reliess heavily on histological assessments (e.g., H&E staining, immunohistochemistry) (Hall et al., 2022). However, these methods require embedding and sectioning, involve labor-intensive procedures, and are inherently end-point measurements. This leads large lots of difficulties in continuous and dynamic observation. Functional assays (such as tyrosinase activity and gene or protein expression) are likewise destructive and do not permit real-time monitoring (Shi et al., 2024; Bass et al., 2017). In contrast, direct observation of three-dimensional pigmented epidermis-on-a-chip models under brightfield microscopy provides a non-invasive morphological evaluation approach. It is easy to perform and enables rapid assessment of melanin distribution and overall model status. Nevertheless, results depend on subjective judgment and lack quantitative precision and standardized comparability. Against this backdrop, the introduction of image-based artificial intelligence (AI) analysis is of particular importance. It can transform subjective image inspection into objective, standardized, and scalable quantitative metrics, sensitively capturing multiscale spatial heterogeneity difficult to discern with the naked eye (Wu et al., 2022; Schmitz et al., 2021). On the other hand, it allows evaluation under label-free brightfield imaging conditions (Shen et al., 2022; Park et al., 2023), thereby facilitating verification of the stability and consistency of the model as a testing platform. However, this process still faces several methodological challenges: first, brightfield images are easily affected by uneven illumination, glare, and out-of-focus artifacts, necessitating color and illumination calibration; second, melanin patches often have fuzzy boundaries and pronounced variations in scale, undermining the comparability of results across wells and time points; and finally, the use of different magnifications may further lower the analytical consistency. The current advancements in deep learning can offer solutions for blurred boundary problems, such as introducing edge-aware branches (Dong et al., 2022), graph convolution (Wang et al., 2024; Liu et al., 2022), and edge-aware loss functions (Zhan and Yang, 2025; Zheng et al., 2020), *etc.* For multi-scale variation problems, researchers have proposed multi-scale dilated (expansion) convolution methods (Wang et al., 2023; Gao et al., 2026) and different meta-learners to capture long-range and short-range dependencies (Yang et al., 2025; Yang et al., 2026; Holail et al., 2025), *etc.*

To achieve the effectively analysis, an artificial intelligence -based evaluation framework was established for pigmented epidermis-on-a-chip models using brightfield images (Figure 1b). Based on our proposed MEM-ViT (Melanin Estimation *via* Vision Transformer) algorithm, precise segmentation of melanin regions was first performed to generate mask images, and subsequently extract four static metrics from these masks to quantitatively characterize the extent of pigment deposition, as well as its spatial scale and optical properties. Furthermore, these metrics were jointly analyzed to enable standardized determination of model quality (“good” vs. “poor”) and the suitability of the model as a testing platform. Experimental results demonstrate that, compared with existing deep learning algorithms, MEM-ViT achieved high-precision segmentation of melanin regions and extraction of their morphological features, and effectively mitigated interference due to the variations in brightfield imaging conditions and culture environments. This work provides a non-invasive, efficient, and reliable technical approach for graded evaluation of pigmented epidermis-on-a-chip models.

## 2 Materials and methods

### 2.1 Cell sources and culture

Normal human keratinocytes (NHKs) and normal human melanocytes (NHMs) were isolated from foreskin tissue obtained from pediatric circumcision procedures. Sample collection complied with relevant laws and institutional ethical guidelines, and written informed consent and approval were obtained from the institutional ethics committee. Cells were isolated by separating the epidermis and dermis using 0.25% (w/v) trypsin at 4 °C overnight. On the following day, basal layer cells were gently scraped from the dermal papillary surface and collected by centrifugation at 200 *g* for 5 min. These procedures were carried out in accordance with Li et al. (2023).

NHKs were seeded onto collagen IV–precoated culture dishes and maintained in the same medium system as described by Li et al. (2023). NHMs were cultured in Medium 254 (Gibco, United States) supplemented with Human Melanocyte Growth Supplement (Gibco, United States). Cells were routinely maintained at 37 °C in a humidified atmosphere of 5% CO_2_, subcultured at 60%–80% confluence, and used within six passages (Li et al., 2023).

When constructing melanocyte-containing systems, keratinocytes and melanocytes were seeded at an approximate ratio of 10:1, and the keratinocyte and melanocyte growth media were mixed at the same 10:1 ratio to prepare the co-culture medium. This formulation has been validated in the same model to support NHK proliferation and differentiation while remaining compatible with NHM co-culture. During the air–liquid interface (ALI) differentiation phase, the calcium concentration in the medium was adjusted to 1.2 mM.

### 2.2 Construction of 3D pigmented epidermis-on-a-chip models

Following the method for constructing 3D pigmented epidermis-on-a-chip models described by Li et al. (2023), keratinocytes and melanocytes were seeded onto PET porous membranes at a total density of approximately 6 × 10^5^ cells per well, with a keratinocyte-to-melanocyte ratio of about 10:1. After 2 days of submerged co-culture, the cultures were transitioned to air–liquid interface (ALI) conditions for 14 days to promote epidermal differentiation and formation of a stratified, melanin-containing epidermis. Detailed procedures, reagents, and equipment are described in Li et al. (2023).

### 2.3 Data acquisition and annotation

Avatarget high-throughput imaging system was used to acquire brightfield images of the pigmented epidermis-on-a-chip models. After several days of culture, images were captured at ×4 and ×10 magnification. For each sample, multiple fields of view were imaged and stitched when necessary to cover the entire well of the plate. To improve image sharpness and avoid defocus, multi-plane 2D images were processed using a focus-stacking algorithm to generate fully focused composite images. The final images were saved in JPG or TIFF format at resolutions of 2,248 × 2,048 or 1,360 × 1,024 pixels. In the semantic segmentation dataset, each brightfield image was manually annotated by two trained experts using the LabelMe software. The annotators delineated, at the pixel level, the boundaries of melanocytes and melanin patches, labeling all visible cells and spheroid structures as foreground (class 1), and all other regions, including the background and culture medium, as background (class 0). Ambiguous regions were carefully reviewed because of the relatively low contrast of brightfield images. If the inter-annotator agreement, measured by the pixel-wise intersection-over-union (IoU), was below 80%, the image was re-evaluated and corrected by a third senior annotator. The final binary masks were exported in PNG format, maintaining a one-to-one correspondence with the original images.

### 2.4 Vision transformer

Vision Transformer (ViT) is a model that introduces the Transformer architecture, originally developed in the field of natural language processing, into computer vision tasks (Dosovitskiy, 2020). Unlike conventional convolutional neural networks (CNNs), which rely on local convolutional kernels to capture spatial features, ViT divides an image into fixed-size patches, flattens them, and encodes them into a one-dimensional sequence. Afterwards, it used a self-attention mechanism to model relationships among patches at a global level, thereby enabling more effective capture of long-range dependencies and global contextual information. Within each Transformer layer, self-attention is computed as:

$$Attention\left(Q,K,V\right)=softmax\left(\frac{QK^{T}}{\sqrt{d_{k}}}\right)V$$

where Q , K , and V denote the query, key, and value matrices derived from the patch embeddings. This global modeling capability allows ViT to exhibit powerful feature extraction performance when trained on large-scale datasets with sufficient computational resources. Vision Transformers show considerable promise in semantic segmentation tasks. Traditional CNN-based models are often limited by their local receptive fields when dealing with complex scenes, whereas Transformer-based architectures can effectively integrate global semantic information during the encoding stage, leading to more accurate segmentation in scenarios with blurred boundaries or subtle inter-class differences (Han et al., 2023). Recently proposed variants, such as TransUNet (Chen et al., 2021) and Swin-UNet (Cao et al., 2022), combine Transformer modules with U-Net–style encoder–decoder architectures and have achieved significant performance gains in medical imaging. We also apply the encoder–decoder architecture to the algorithms in this paper. At each decoding stage i , the upsampled feature map $F_{i}^{up}$ is fused with the corresponding skip feature $F_{i}^{skip}$ as:

$$F_{i}=\emptyset \left(F_{i}^{up}⊕\;F_{i}^{skip}\right)$$

where ∅ denotes channel-wise concatenation and ⊕ represents convolutional transformations followed by normalization and non-linear activation.

### 2.5 Model optimization

To improve segmentation performance on low-contrast brightfield images, the following optimization strategies were adopted during training. As deep learning models are supervised methods, they require a large number of data samples to adequately tune model parameters. However, manually annotating melanin regions in images of the pigmented epidermis-on-a-chip models is time-consuming and labor-intensive. Therefore, to mitigate overfitting in the small-sample setting, data augmentation was conducted to increase both the size and diversity of the training set.

Typical augmentation techniques were applied, including random cropping, geometric transformations (e.g., rotation, translation, and flipping), adjustments of color, intensity, and contrast, as well as non-rigid image transformations (e.g., elastic deformations). The model was optimized using a composite loss function, defined as a weighted sum of binary cross-entropy (BCE) loss and Dice loss. In the BCE term, the positive class was reweighted (weighting factor 0.5) to alleviate the dominance of abundant background pixels (Li et al., 2024; Zhao et al., 2020). The Dice loss simultaneously optimizes pixel-wise classification and the overall overlap between predicted and ground-truth shapes.

Adam optimizer (initial learning rate 1 × 10^−3^, β_1_ = 0.9, β_2_ = 0.99) was used and a 50% learning rate decay was applied if the validation loss did not decrease for 10 consecutive epochs. In addition, dropout layers with a rate of 0.5 were inserted at the end of the encoder and the beginning of the decoder to further suppress overfitting (Srivastava et al., 2014).

$$L_{loss}=\lambda L_{BCE}+\left(1-\lambda \right)L_{Dice}$$

### 2.6 Model training and evaluation

We trained the model on a workstation equipped with four NVIDIA RTX 3090 GPUs (11 GB memory each). The dataset was split on a per-sample basis into training (50%), validation (20%), and test (30%) sets to ensure that images from different magnifications were evenly distributed across the three subsets. The batch size was set to 8, and the maximum number of training epochs was 200, with early stopping applied when the validation performance no longer improved. During training, the raw images and their corresponding binary masks were used as input–label pairs. The input images were normalized before feeding into the network, and the resulting probability maps were binarized using a threshold of 0.45 to generate the predicted segmentation masks.

To assess the segmentation performance of the trained model, comparison between model predictions and ground-truth labels is typically quantified using four basic concepts. True positives (TP) denote the number of pixels correctly identified by the model as belonging to the target class. False positives (FP) refer to pixels that are incorrectly classified as the target class when they actually do not. True negatives (TN) represent pixels correctly identified as background, whereas false negatives (FN) correspond to target pixels that the model fails to detect, i.e., pixels that truly belong to the target class but are misclassified as background. Based on these quantities, several commonly used evaluation metrics can be computed. Accuracy (Acc) measures the overall correctness of pixel-wise classification and reflects the model’s global segmentation performance. The Dice similarity coefficient (DSC, also known as the F1-score) evaluates the overlap between predicted and ground-truth regions, placing greater emphasis on the consistency of the target region. The intersection-over-union (IoU) quantifies the ratio between the intersection and union of the predicted and ground-truth masks and is one of the most widely used metrics for assessing semantic segmentation performance. In summary, Acc reflects overall accuracy, DSC emphasizes overlap consistency, and IoU provides a more stringent assessment of the quality of the predicted regions (Cheng et al., 2021; Zou et al., 2004; Minaee et al., 2021).

$$IOU=\frac{TP}{TP+FP+FN}$$

$$Acc=\frac{TP+TN}{TP+FP+TN+FN}$$

$$DSC=\frac{2\times TP}{2\times TP+FP+FN}$$

### 2.7 Evaluation indicators

Once the segmentation masks was obtained from the model, quantitative analysis was performed to evaluate the characteristics of melanin distribution and to interpret the biological or pharmacological relevance of the segmentation results. The primary evaluation metrics include the area-related, optical, and spatial distribution features of melanin patches. The specific definitions and descriptions of these metrics are provided below.

#### 2.7.1 Total area of melanin patches:

$$A_{total}=\sum_{i=1}^{N}A_{i}$$

Here, $A_{i}$ denotes the area of the i−th melanin patch, and N is the total number of patches. This metric reflects the overall level of melanin deposition in the sample. A larger total melanin area indicates more severe melanin accumulation, whereas a marked reduction suggests that melanogenesis is inhibited or that a pronounced depigmenting effect has been achieved.

#### 2.7.2 Average area of melanin patches

$$A_{mean}=\frac{1}{N}\;\sum_{i=1}^{N}A_{i}$$

Mean area ( $A_{mean}$ ) is used to evaluate the average size of individual melanin deposition sites and can indicate whether melanogenesis tends to be locally concentrated or diffusely distributed. A larger $A_{mean}$ suggests a tendency for melanin to form larger aggregated patches, whereas a smaller $A_{mean}$ ​ may indicate more dispersed melanin granules and lower melanogenic activity.

#### 2.7.3 Melanin patch light transmittance

$$T=\frac{I}{I_{0}}\times 100\%$$

Here, I denotes the mean light intensity within the melanin patch region, and $I_{0}$ represents the mean light intensity in the background or control region. Transmittance T is used to quantify the degree of light absorption by melanin-containing areas. Greater melanin deposition corresponds to lower transmittance since melanin strongly absorbs light. This metric can be used to evaluate the efficacy of skin-whitening or anti-melanogenic agents: an increase in T indicates enhanced light transmittance of the skin or tissue and a reduction in melanin content.

#### 2.7.4 Optical density of melanin patches

$$OD=-\log_{10}\left(\frac{I}{I_{0}}\right)$$

Here, I and $I_{0}$ are defined as above, and a higher OD value indicates more intense melanin deposition. Optical density ( OD ) is an optical parameter that characterizes the concentration of melanin deposition and is commonly used in the analysis of microscopic images and histological sections. An increase in OD reflects stronger light absorption and a higher melanin concentration in the corresponding region. In general, OD is positively correlated with melanin content.

#### 2.7.5 Composite score formula

We aimed to aggregate multiple raw metrics (total patch area ( $A_{total}$ ), mean patch area ( $A_{mean}$ ), transmittance ( T ), and optical density ( OD )) into a single composite score ranging from 0 to 100, where higher scores indicate more severe melanin deposition. To this end, a min–max linear transformation was applied to rescale each set of measurements to the specified range (e.g., ([0, 100])).

$$x^{\prime }=\frac{x-x_{\min}}{x_{\max}-x_{\min}}\times \left(b-a\right)+a$$

Here, x denotes the original data, $x_{\min}$ and $x_{\max}$ are the minimum and maximum values of the data, respectively, and a and b are the lower and upper bounds of the target range (set to 0 and 100 in this study). *x* ^′^ denotes the normalized result.

The composite score is obtained by linearly combining the above rescaled metrics as follows:

$$score=w_{Atotal}*A_{total}^{\prime }+w_{Amean}*A_{mean}^{\prime }+w_{T}*T^{\prime }+w_{OD}*OD^{\prime }$$

Here, $w_{\text{Atotal}}$ = 0.35, $w_{\text{Amean}}$ = 0.10, $w_{T}$ = 0.15, and $w_{\text{OD}}$ = 0.30. *A′_total_, A′_mean_, T′, and OD′* represent the normalized total patch area, mean patch area, transmittance,and optical density, respectively. Since *A′_total_* and *OD′* are typically the primary indicators, they are assigned higher weights. In contrast, *A′_mean_* was used mainly to characterize the distribution pattern, and thus its weight was kept lower to avoid redundant emphasis on area-related information.

## 3 Results

### 3.1 Formation of the 3D pigmented epidermis-on-a-chip model and AI-based analysis

**FIGURE 2:**
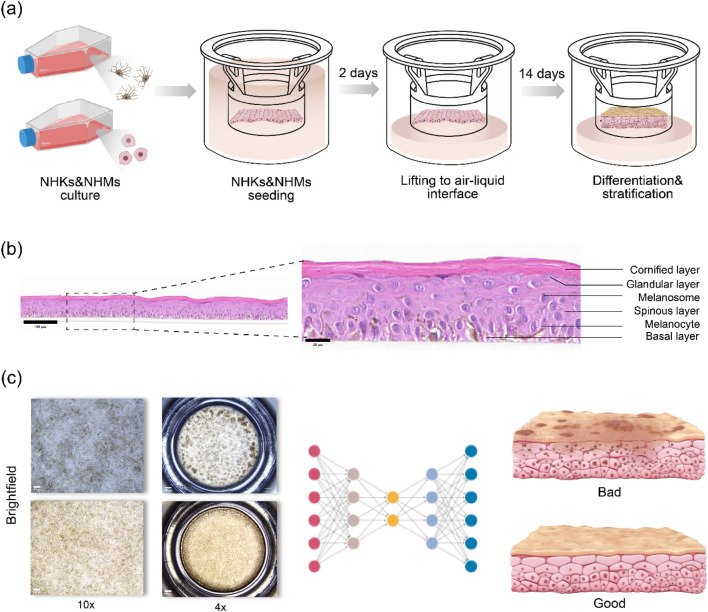
Construction of the pigmented epidermis-on-a-chip model and evaluation metrics: **(a)** Schematic illustration of submerged co-culture of normal human keratinocytes and melanocytes on the chip, followed by proliferation and differentiation of keratinocytes under air–liquid interface (ALI) conditions to form a stratified epidermis; **(b)** H&E staining of the pigmented epidermis-on-a-chip model after 2 weeks of ALI culture, with a magnified view of the local epidermal structure; and **(c)** Brightfield images of the pigmented epidermis-on-a-chip model are input into the algorithm, which evaluates the epidermal model (good vs. poor) based on the defined quantitative metrics.

In this study, the three-dimensional pigmented epidermis-on-a-chip model developed under air–liquid interface (ALI) culture formed a well-defined stratified epidermal structure after 2 weeks (Figures 2a,b). Melanocytes were clearly localized to the basal layer, and melanin was efficiently synthesized and transferred to the overlying keratinocytes, thereby recapitulating the *in vivo* pattern of epidermal melanin deposition (Figure 2b). To assess model quality, samples were categorized into two groups (“good” and “poor”) based on morphological features observed in microscopic images.

**“Good”** models: Pigmentation appeared as fine, discrete micro-spots with an overall homogeneous distribution (Figure 2c). No large confluent patches were observed at low magnification and only delicate and uniform microtextures were present at higher magnification. This morphology suggests stable melanogenesis and efficient melanin transfer to keratinocytes, with well-coupled spatial organization. Samples with these characteristics were defined as “good” and are recommended as suitable testing platforms for skincare products, cosmetics, and pharmacological agents.

**“Poor”** models: Multiple dark, island-like pigmented foci were present and frequently fused into larger patches (Figure 2c), forming extensive areas at low magnification. Pronounced edge effects or ring-shaped inhomogeneities were often observed (e.g., the peripheral region markedly darker than the center, or *vice versa*). At high magnification, the patch size distribution was heterogeneous, and local textures appeared coarse. Such features indicate spatial disequilibrium in melanin production and transfer, with substantial variability both within batches and between positions. These samples are classified as “poor” and are not recommended as stable testing platforms.

To more comprehensively evaluate the quality of the pigmented epidermis-on-a-chip model, the outputs obtained after algorithmic segmentation of melanin patches were analyzed. A set of morphological and optical parameters were established as evaluation metrics, including: (1) total melanin patch area to reflect the overall level of melanin deposition in the model; (2) mean melanin patch area to characterizes the uniformity of melanin deposition and the degree of pigment aggregation; (3) melanin patch transmittance to assess the impact of melanin on light penetration based on image grayscale values or optical measurements, assesses; and (4) melanin patch optical density calculated from grayscale intensity to quantitatively describe the concentration and depth of melanin deposition. These metrics were subsequently rescaled and linearly combined to obtain a composite evaluation score. Joint analysis of the above indicators not only enables a macroscopic assessment of the overall pigmentation of the model, but also reveals the spatial distribution and optical characteristics of melanin deposition at a microscopic level. This provides a systematic basis for comparing differences in melanogenesis under different culture conditions, external stimuli, or pharmacological treatments.

To enable comprehensive evaluation of the model, full-well images at ×4 magnification was acquired as the primary quantitative dataset, whereas images at ×10 magnification were used only as morphological corroboration (to examine the presence of pigment aggregates and textural features). A total of 30 pigmented epidermis-on-a-chip models were included in this study. During image acquisition, approximately 1000 brightfield images were collected in total, because multiple raw fields of view were acquired for each model at different magnifications and stitched when necessary to cover the entire well. For quantitative analysis, each full-well composite image corresponded to one epidermal chip and served as the primary input for MEM-ViT analysis.

### 3.2 Deep learning–based melanin detection method

**FIGURE 3:**
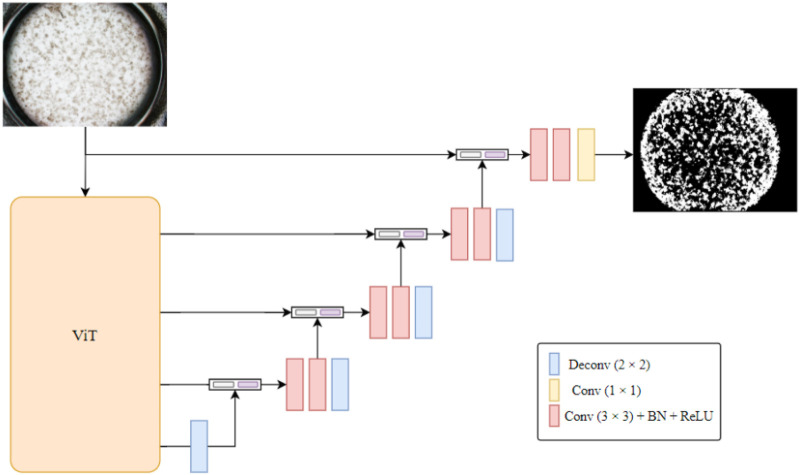
Overall architecture of the MEM-ViT algorithm. The algorithm utilizes a Vision Transformer as the backbone network to extract features from the input image, and incorporates a “U”-shaped concatenation structure to output the melanin area.

Within the overall methodological framework described in Section 2, the proposed MEM-ViT network serves as the core component for melanin detection. The overall architecture is shown in Figure 3. Thenetwork consists of three main components: (1) a ViT encoder (Dosovitskiy, 2020), (2) a multi-branch decoder, and (3) a post-processing pipeline. In this network, a Vision Transformer (ViT) was employed as the encoder for 3D epidermal images, leveraging the self-attention mechanism to directly model global relationships among voxel patches and thereby overcome the local receptive field limitations of conventional convolutional neural networks. The features produced by the encoder were propagated to the upsampling decoder *via* skip connections, enabling fusion of high-level semantic information with low-level details. Inspired by U-Net (Ronneberger et al., 2015), five skip connections were design in the decoder. The first skip connection originates from the raw input image and performs feature extraction using two 3 × 3 convolutional layers with batch normalization and ReLU activation. The remaining four skip connections extract tokens from intermediate ViT layers that are reshaped into 3D tensors and then fed into the decoder; each feature map is further processed by two convolutional layers to preserve multi-scale contextual information. Deep features are progressively upsampled through a series of transposed convolution layers, which double the spatial resolution in all three directions at each stage, followed by convolutions to adjust the channel dimension. At each decoding stage, the features are fused with those from the corresponding skip connection to ensure effective integration of multi-scale information, thereby improving the segmentation accuracy of the epidermal structure. Finally, the resulting segmentation mask undergoes post-processing to exactly match the resolution of the input 3D pigmented epidermis-on-a-chip models.

### 3.3 Model performance evaluation

**FIGURE 4:**
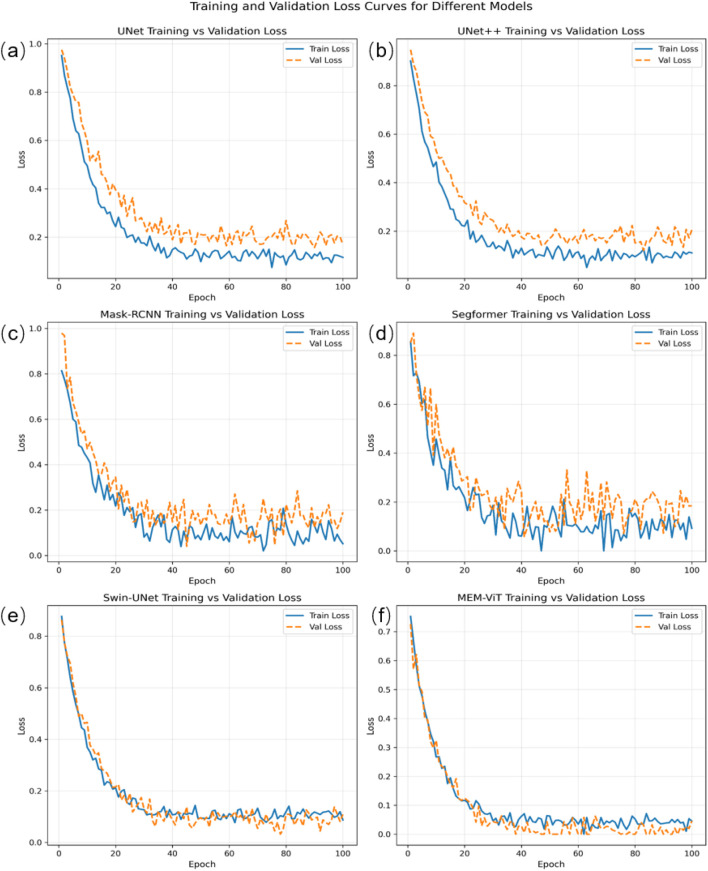
Training loss curves of different models on the training and validation sets. **(a–f)** Comparison of training and validation losses for six classical semantic segmentation models on the 3D pigmented epidermis-on-a-chip dataset.

Quantitative assessment of the morphological features of 3D pigmented epidermis-on-a-chip models under brightfield imaging—such as melanin distribution, integrity of the epidermal structure, and other morphological parameters—constitutes one of the most direct and critical evaluation criteria in skin pigmentation research and drug screening. Unlike conventional two-dimensional cell images, which typically exhibit clear and stable monolayer structural features, brightfield imaging of 3D pigmented epidermis-on-a-chip models is influenced by multiple factors, including the culture system, tissue thickness, imaging depth, illumination conditions, and pharmacological interventions. Therefore, images acquired under different experimental conditions and magnifications often exhibit pronounced imaging artifacts. This poses serious challenges for both traditional image processing methods and current deep learning algorithms. These artifacts mainly include local occlusion and illumination inhomogeneity caused by melanin deposition, defocus and signal attenuation induced by imaging depth, morphological variations arising from different culture conditions, as well as interlayer tissue overlap and blurred boundaries. In summary, these complex factors make the automatic identification and segmentation of melanin structures within the epidermal layer under brightfield imaging highly uncertain and challenging. To systematically evaluate the performance of different algorithms in such complex scenarios, a brightfield image dataset of 3D pigmented epidermis-on-a-chip models was constructed and consisted of approximately 1000 images of pigmented epidermal models. The dataset was randomly divided into 50% for training, 20% for validation,and 30% for testing. In our experiments, the proposed method was compared with several representative cell and tissue segmentation architectures, including U-Net (Ronneberger et al., 2015), U-Net++ (Zhou et al., 2020), SegFormer (Xie et al., 2021), and Mask R-CNN (Johnson, 2018). Each model was adapted and optimized for our task and trained for 100 epochs under the same training protocol (Figure 4). Mask R-CNN and SegFormer exhibited relatively large oscillations in the training loss, whereas the other models showed smoother convergence; our model achieved the fastest and most stable convergence. Furthermore, to quantitatively evaluate melanin segmentation performance, we employed three metrics—IoU, DSC, and Acc—to assess the segmentation accuracy of six models, including our MEM-ViT (Table 1). The results showed that our method achieved improvements of 2.4%, 4.3%, and 2.2% in IoU, DSC, and Acc, respectively, compared with mainstream algorithms. Even under conditions of uneven illumination, tissue overlap, and complex morphology, our approach maintained high robustness and accuracy. Table 2 presents a comparative analysis of computational efficiency at a 512 × 512 resolution. SegFormer demonstrates superior real-time performance with a minimum latency of 9.3 ms, attributed to its efficient hierarchical design. Conversely, Mask R-CNN incurs the highest computational cost (182.4G FLOPs) because of its multi-scale feature pyramid network and the dense per-proposal computations in the RPN and mask/box heads. Notably, Mask-RCNN exhibits the maximum latency (45.2 ms), reflecting the architectural overhead of its two-stage pipeline despite moderate FLOPs. Although MEM-ViT possess larger parameter footprints, they maintain competitive inference speeds, suggesting that ViT-B backbones offer an optimal trade-off between model capacity and throughput for this task.

**TABLE 1:** Comparison of melanin segmentation performance across different models.

Methods	​	Metrics	​
	IoU (%)	DSC (%)	Acc (%)
U-Net	65.2	76.8	88.1
U-Net++	69.5	81.4	89.5
Mask-RCNN	63.7	75.1	87.6
SegFormer	60.8	72.4	86.3
Swin-UNet	71.8	80.3	90.2
MEM-ViT	**74.2**	**84.6**	**92.4**

**TABLE 2:** Performance comparison of various architectures at the same input resolution.

Methods	Backbone	Params (M)	FLOPS (G)	Inference time (ms)
U-Net	Standard CNN	31.0	68.9	18.4
U-Net++	Standard CNN	39.5	82.2	32.8
Mask-RCNN	ResNet50-FPN	44.3	182.4	45.2
SegFormer	MiT-B0	24.7	72.6	9.3
Swin-UNet	Swin-B	88.2	116.2	22.6
MEM-ViT	ViT-B	89.5	132.6	26.1

### 3.4 Experimental evaluation

**FIGURE 5:**
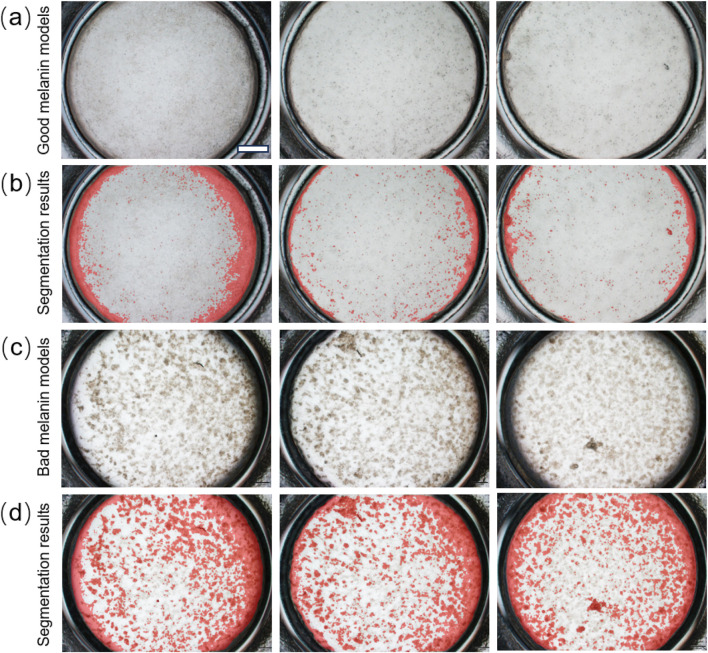
Visualization of “good” and “poor” pigmented epidermis-on-a-chip models and their corresponding segmentation results: **(a)** Brightfield image of a “good” pigmented epidermis-on-a-chip model. Scale bar: 1 mm; **(b)** Segmentation result of the “good” model produced by the proposed algorithm; **(c)** Brightfield image of a “poor” pigmented epidermis-on-a-chip model; and **(d)** Segmentation result of the “poor” model produced by the proposed algorithm. In **(b, d)**, the red overlay represents the AI-predicted segmentation mask used to visualize melanin patches rather than melanin intensity. The uniform peripheral red area reflects a cell-free blank plate background exposed by centripetal contraction during culture and should not be interpreted as homogeneous pigmentation.

Prediction and visualization were performed on samples from the test set to visually demonstrate the performance of the proposed model on the semantic segmentation of 3D pigmented epidermis-on-a-chip models under brightfield imaging, and to further validate the accuracy of the AI-based composite evaluation method in discriminating between “good” and “poor” models (Figures 5a,b). The first row shows brightfield microscopic images of well-performing pigmented epidermis-on-a-chip models, characterized by an intact epidermal structure and homogeneous pigment distribution that closely resembles native skin tissue. The second row presents the corresponding segmentation results, in which the algorithm exhibits high consistency and accuracy in boundary localization and region identification, effectively capturing the spatial distribution of melanin. The third row displays brightfield images of poorly performing models, marked by uneven pigment distribution and large island-like pigmented patches. The fourth row shows the corresponding segmentation visualizations, where the algorithm effectively distinguishes melanin islands of different sizes.

To further validate the reliability and generalization capability of the proposed AI-based method for objective evaluation of pigmented epidermal models, extensibility tests were conducted under different experimental conditions and across multiple sample batches. By assessing model performance on an independent test set not used during training and analyzing the consistency of responses across multiple feature-based indicators, we aimed to verify the robustness of the model under varying image quality, illumination conditions, and melanin distribution patterns.

**FIGURE 6:**
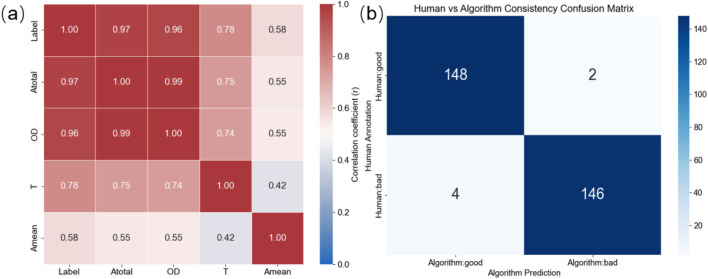
Evaluation of metric correlations and algorithm accuracy: **(a)** Correlation heatmap between the proposed evaluation metrics ( $A_{total}$ , OD , T , $A_{mean}$ ) and the *labels (good or bad)*; and **(b)** Confusion matrix comparing manual annotations and algorithmic predictions on the test set.

Figure 6a shows the Pearson correlation heatmap between the multidimensional evaluation metrics proposed in this study ( $A_{total}$ , OD , T , $A_{mean}$ ) and the manually assigned labels *(Label, good/bad)*. The results indicate that $A_{total}$ and OD exhibit a strong positive correlation with the manual labels (r > 0.95), suggesting that these two parameters effectively capture the overall pigment load and optical density of the samples and are key variables for distinguishing between high- and low-quality models. T metric shows a moderate correlation with the manual labels (r ≈ 0.78), suggesting that this indicator to some extent reflects transmittance characteristics associated with melanin deposition and provides useful reference for model quality discrimination. In contrast, $A_{mean}$ displays a relatively weaker correlation with the labels (r ≈ 0.58), but still shows a consistent trend, indicating that this parameter provides auxiliary information in characterizing the uniformity of pigment distribution. Overall, the high correlations among these metrics support the consistency and rationality of the selected features in describing the state of the pigmented epidermal models.

Figure 6b presents the confusion matrix illustrating the agreement between manual annotations and algorithmic predictions. The results showed that the method achieved high discriminative accuracy for both ‘Good’ and ‘Bad’ samples: among the samples manually classified as ‘Good,’ 148 cases were consistent with the algorithm-based classification,with only 2 misclassified; among the samples manually classified as ‘Bad,’ 146 were correctly classified and 4 were misclassified. The overall agreement reached 98%, indicating that the evaluation system constructed based on MEM-ViT segmentation results and multi-indicator fusion exhibited high reliability and stable discriminative performance in distinguishing high-quality from low-quality pigmented epidermis-on-a-chip models. These findings further support the feasibility and scientific validity of the multi-metric feature fusion–based quantitative evaluation approach for automated analysis of brightfield images.

## 4 Discussion

In this study, an AI-assisted quantitative evaluation framework was established for a pigmented epidermis-on-a-chip model to objectively assess melanin distribution under bright-field imaging. By integrating a 3D pigmented epidermal culture with an automated image-analysis pipeline, the system can classify overall pigmentation outcomes and simultaneously extract multiple quantitative parameters describing the area, intensity and spatial distribution of melanin. The AI-based classification reached 98% agreement with expert annotations, supporting its reliability as an objective readout for pigmentation in epidermis-on-a-chip platforms and laying the groundwork for subsequent applications such as efficacy testing and mechanistic studies.

Previous studies evaluating pigmentation in skin equivalents or reconstructed epidermal models mainly relied on qualitative or semi-quantitative approaches such as gross visual inspection, histological staining (e.g., Fontana–Masson) and immunohistochemistry or immunofluorescence of melanogenesis-related markers (Hall et al., 2022; Nissan et al., 2011). These methods require tissue processing and sectioning, are time-consuming and costly, and are intrinsically destructive, which precludes longitudinal assessment on the same sample and limits their suitability for high-throughput applications (Benito-Martínez et al., 2020). Even with the introduction of digital image analysis, it is often limited to simple readings, such as the percentage of pixels corresponding to melanin in manually selected epidermal regions or the average intensity of user-defined areas. This leads to considerable inter-operator variability and challenges in standardizing quantitative criteria across laboratories (Miot et al., 2012; Pena et al., 2022). By contrast, our AI-based framework operates directly on bright-field images of intact pigmented epidermis-on-a-chip, eliminating the need for additional labeling or invasive sampling and thus enabling non-invasive, low-cost, and scalable assessment of melanin distribution. Compared with these traditional workflows, the automated pipeline markedly improves analytical efficiency, reduces dependence on subjective human scoring, and facilitates reproducible, standardized evaluation of pigmentation for large-scale experiments and screening studies.

Another advantage of our approach is that it provides a multidimensional quantitative description of pigmentation rather than a single aggregated score. From bright-field images, the model outputs area-related, optical-density and spatial-distribution features of melanin, enabling a more nuanced and biologically informative characterization of pigmentation patterns (Benito-Martínez et al., 2020; Pena et al., 2022). Compared with AI methods that primarily generate segmentation masks or global hyperpigmentation severity scores and typically report only standard metrics such as IoU, DSC or accuracy (Kojima et al., 2021; Zhang et al., 2025; Draelos et al., 2025), our ViT-based segmentation model, when benchmarked against U-Net, UNet++, SegFormer and Mask R-CNN, achieved the highest IoU, DSC and pixel-level accuracy on both the training and independent test sets, supporting its robustness and generalizability for automated analysis of pigmented epidermis-on-a-chip images (Ronneberger et al., 2015; Zhou et al., 2020; Xie et al., 2021; Johnson, 2018).

This study still has several limitations, as the framework was trained on a relatively small dataset and relies only on static endpoint images. Future work will expand data diversity and incorporate dynamic time-series modeling by combining imaging features with temporal changes in melanin production and redistribution under different stimuli to more closely approximate the native skin microenvironment. Such a spatiotemporal extension would further increase the utility of the model for drug screening, studies of skin-whitening mechanisms and the assessment of pathological pigmentation states.

## 5 Conclusion

In summary, the AI-driven quantitative evaluation framework proposed demonstrates high accuracy, repeatability, and biological interpretability in the brightfield image analysis of three-dimensional pigmented epidermis-on-a-chip models. By combining semantic segmentation with multi-metric feature fusion, this approach enables an objective and standardized assessment of melanin distribution. Future studies will further extend this framework by incorporating time-series analysis to achieve dynamic monitoring of melanogenesis and melanin metabolism. This is expected to provide new technical support for drug efficacy evaluation and studies of skin-whitening mechanisms.

## Data Availability

The datasets presented in this study can be found in online repositories. The names of the repository/repositories and accession number(s) can be found in the article/supplementary material.
